# The Human Phenotype Ontology in 2024: phenotypes around the world

**DOI:** 10.1093/nar/gkad1005

**Published:** 2023-11-11

**Authors:** Michael A Gargano, Nicolas Matentzoglu, Ben Coleman, Eunice B Addo-Lartey, Anna V Anagnostopoulos, Joel Anderton, Paul Avillach, Anita M Bagley, Eduard Bakštein, James P Balhoff, Gareth Baynam, Susan M Bello, Michael Berk, Holli Bertram, Somer Bishop, Hannah Blau, David F Bodenstein, Pablo Botas, Kaan Boztug, Jolana Čady, Tiffany J Callahan, Rhiannon Cameron, Seth J Carbon, Francisco Castellanos, J Harry Caufield, Lauren E Chan, Christopher G Chute, Jaime Cruz-Rojo, Noémi Dahan-Oliel, Jon R Davids, Maud de Dieuleveult, Vinicius de Souza, Bert B A de Vries, Esther de Vries, J Raymond DePaulo, Beata Derfalvi, Ferdinand Dhombres, Claudia Diaz-Byrd, Alexander J M Dingemans, Bruno Donadille, Michael Duyzend, Reem Elfeky, Shahim Essaid, Carolina Fabrizzi, Giovanna Fico, Helen V Firth, Yun Freudenberg-Hua, Janice M Fullerton, Davera L Gabriel, Kimberly Gilmour, Jessica Giordano, Fernando S Goes, Rachel Gore Moses, Ian Green, Matthias Griese, Tudor Groza, Weihong Gu, Julia Guthrie, Benjamin Gyori, Ada Hamosh, Marc Hanauer, Kateřina Hanušová, Yongqun (Oliver) He, Harshad Hegde, Ingo Helbig, Kateřina Holasová, Charles Tapley Hoyt, Shangzhi Huang, Eric Hurwitz, Julius O B Jacobsen, Xiaofeng Jiang, Lisa Joseph, Kamyar Keramatian, Bryan King, Katrin Knoflach, David A Koolen, Megan L Kraus, Carlo Kroll, Maaike Kusters, Markus S Ladewig, David Lagorce, Meng-Chuan Lai, Pablo Lapunzina, Bryan Laraway, David Lewis-Smith, Xiarong Li, Caterina Lucano, Marzieh Majd, Mary L Marazita, Victor Martinez-Glez, Toby H McHenry, Melvin G McInnis, Julie A McMurry, Michaela Mihulová, Caitlin E Millett, Philip B Mitchell, Veronika Moslerová, Kenji Narutomi, Shahrzad Nematollahi, Julian Nevado, Andrew A Nierenberg, Nikola Novák Čajbiková, John I Nurnberger, Soichi Ogishima, Daniel Olson, Abigail Ortiz, Harry Pachajoa, Guiomar Perez de Nanclares, Amy Peters, Tim Putman, Christina K Rapp, Ana Rath, Justin Reese, Lauren Rekerle, Angharad M Roberts, Suzy Roy, Stephan J Sanders, Catharina Schuetz, Eva C Schulte, Thomas G Schulze, Martin Schwarz, Katie Scott, Dominik Seelow, Berthold Seitz, Yiping Shen, Morgan N Similuk, Eric S Simon, Balwinder Singh, Damian Smedley, Cynthia L Smith, Jake T Smolinsky, Sarah Sperry, Elizabeth Stafford, Ray Stefancsik, Robin Steinhaus, Rebecca Strawbridge, Jagadish Chandrabose Sundaramurthi, Polina Talapova, Jair A Tenorio Castano, Pavel Tesner, Rhys H Thomas, Audrey Thurm, Marek Turnovec, Marielle E van Gijn, Nicole A Vasilevsky, Markéta Vlčková, Anita Walden, Kai Wang, Ron Wapner, James S Ware, Addo A Wiafe, Samuel A Wiafe, Lisa D Wiggins, Andrew E Williams, Chen Wu, Margot J Wyrwoll, Hui Xiong, Nefize Yalin, Yasunori Yamamoto, Lakshmi N Yatham, Anastasia K Yocum, Allan H Young, Zafer Yüksel, Peter P Zandi, Andreas Zankl, Ignacio Zarante, Miroslav Zvolský, Sabrina Toro, Leigh C Carmody, Nomi L Harris, Monica C Munoz-Torres, Daniel Danis, Christopher J Mungall, Sebastian Köhler, Melissa A Haendel, Peter N Robinson

**Affiliations:** The Jackson Laboratory for Genomic Medicine, Farmington, CT, USA; Semanticly, Athens, Greece; The Jackson Laboratory for Genomic Medicine, Farmington, CT, USA; Rare Disease Ghana Initiative, Accra, Ghana; The Jackson Laboratory, Bar Harbor, ME, USA; Center for Craniofacial and Dental Genetics, Department of Oral and Craniofacial Sciences, School of Dental Medicine, University of Pittsburgh, Pittsburgh, PA, USA; Harvard Medical School, Boston, MA, USA; Shriners Children's Northern California, Sacramento, CA, USA; National Institute of Mental Health, Klecany, Czech Republic; Renaissance Computing Institute, University of North Carolina, Chapel Hill, NC 27517, USA; Rare Care Centre, Perth Children's Hospital, Perth, Australia; The Jackson Laboratory, Bar Harbor, ME, USA; Deakin University, IMPACT - the Institute for Mental and Physical Health and Clinical Translation, School of Medicine, Barwon Health, Geelong, Australia; Department of Psychiatry, University of Michigan, Ann Arbor, MI, USA; Department of Psychiatry and Behavioral Sciences, UCSF Weil Institute for Neuroscience, San Francisco, CA, USA; The Jackson Laboratory for Genomic Medicine, Farmington, CT, USA; Department of Pharmacology and Toxicology, University of Toronto, Toronto, ON, Canada; Nostos Genomics GmbH, Berlin, Germany; St. Anna Children's Cancer Research Institute (CCRI), Vienna, Austria; Institute of Health Information and Statistics of the Czech Republic, Prague, Czech Republic; Department of Biomedical Informatics, Columbia University Irving Medical Center, NY, NY, USA; Simon Fraser University, Burnaby, BC, Canada; Division of Environmental Genomics and Systems Biology, Lawrence Berkeley National Laboratory, Berkeley, CA 94720, USA; The Jackson Laboratory for Genomic Medicine, Farmington, CT, USA; Division of Environmental Genomics and Systems Biology, Lawrence Berkeley National Laboratory, Berkeley, CA 94720, USA; College of Public Health and Human Sciences, Oregon State University, Corvallis, OR 97331, USA; Schools of Medicine, Public Health, and Nursing, Johns Hopkins University, Baltimore, MD 21287, USA; UDISGEN (Dysmorphology and Genetics Unit), 12 de Octubre Hospital, Madrid, Spain; Department of Clinical Research, Shriners Hospitals for Children, Montreal, Quebec, Canada; Shriners Children's Northern California, Sacramento, CA, USA; Département I&D, AP-HP, Banque Nationale de Données Maladies Rares, Paris, France; European Bioinformatics Institute (EMBL-EBI), Wellcome Genome Campus, Hinxton CB10 1SD, UK; Department of Human Genetics, Donders Institute for Brain, Cognition and Behaviour, Radboud University Medical Center, Nijmegen, Netherlands; Tranzo, TSB, Tilburg University, Netherlands; Department of Psychiatry and Behavioral Sciences, Johns Hopkins University School of Medicine, Baltimore, MD 21287, USA; Department of Pediatrics, Dalhousie University, Halifax, NS, Canada; Fetal Medicine Department, Armand Trousseau Hospital, Sorbonne University, GRC26, INSERM, Limics, Paris, France; Department of Psychiatry, University of Michigan, Ann Arbor, MI, USA; Department of Human Genetics, Donders Institute for Brain, Cognition and Behaviour, Radboud University Medical Center, Nijmegen, Netherlands; St Antoine Hospital, Reference Center for Rare Growth Endocrine Disorders, Sorbonne University, AP-HP, INSERM, US14 - Orphanet, Plateforme Maladies Rares, Paris, France; Massachusetts General Hospital, Boston, MA, USA; Department of Immunology, GOS Hospital for Children NHS Foundation Trust, University College London, London, UK; Department of Biomedical Informatics, University of Colorado Anschutz Medical Campus, Aurora, CO 80045, USA; INSERM, US14 - Orphanet, Plateforme Maladies Rares, Paris, France; Bipolar and Depressive Disorders Unit, Institute of Neuroscience, Hospital Clinic, University of Barcelona, IDIBAPS, CIBERSAM, Barcelona, Catalonia, Spain; Addenbrooke's Hospital, Cambridge University Hospitals, Cambridge, UK; Department of Psychiatry, Feinstein Institutes for Medical Research, Northwell Health, Manhasset, NY, USA; Neuroscience Research Australia, Sydney, NSW, Australia; School of Medicine, Johns Hopkins University, Baltimore, MD 21287, USA; Immunology, NIHR Great Ormond Street Hospital BRC, London, UK; Department of Obstetrics and Gynecology, Columbia University Irving Medical Center, New York, NY, USA; Department of Psychiatry and Behavioral Sciences, Johns Hopkins University School of Medicine, Baltimore, MD 21287, USA; National Institute of Allergy and Infectious Diseases, National Institutes of Health, Bethesda, MD 20892, USA; SNOMED International, London W2 6BD, UK; Department of Pediatrics, Dr. von Hauner Children's Hospital, University Hospital, LMU Munich, German center for Lung research (DZL), Munich, Germany; Rare Care Centre, Perth Children's Hospital, Perth, Australia; Chinese HPO Consortium, Beijing, China; Department of Structural and Computational Biology, University of Vienna; Max Perutz Labs, Vienna, Austria; Khoury College of Computer Sciences, Northeastern University, Boston, MA, USA; Department of Genetic Medicine, Johns Hopkins University School of Medicine, Baltimore, MD 21287, USA; INSERM, US14 - Orphanet, Plateforme Maladies Rares, Paris, France; Institute of Health Information and Statistics of the Czech Republic, Prague, Czech Republic; University of Michigan Medical School, Ann Arbor, MI, USA; Division of Environmental Genomics and Systems Biology, Lawrence Berkeley National Laboratory, Berkeley, CA 94720, USA; Neurology, Children's Hospital of Philadelphia, Philadelphia, PA 19104, USA; Institute of Health Information and Statistics of the Czech Republic, Prague, Czech Republic; Khoury College of Computer Sciences, Northeastern University, Boston, MA, USA; Chinese HPO Consortium, Beijing, China; University of Colorado Anschutz Medical Campus, Aurora, CO 80045, USA; William Harvey Research Institute, Queen Mary University of London, London, UK; Chinese HPO Consortium, Beijing, China; Neurodevelopmental and Behavioral Phenotyping Service, National Institute of Mental Health, Bethesda, MD, USA; Department of Psychiatry, University of British Columbia, Vancouver, BC, Canada; Department of Psychiatry and Behavioral Sciences, UCSF Weil Institute for Neuroscience, San Francisco, CA, USA; Department of Pediatrics, Dr. von Hauner Children's Hospital, University Hospital, LMU Munich, German center for Lung research (DZL), Munich, Germany; Department of Human Genetics, Donders Institute for Brain, Cognition and Behaviour, Radboud University Medical Center, Nijmegen, Netherlands; University of Colorado Anschutz Medical Campus, Aurora, CO 80045, USA; William Harvey Research Institute, Queen Mary University of London, London, UK; Immunology, NIHR Great Ormond Street Hospital BRC, London, UK; Department of Ophthalmology, University Clinic Marburg - Campus Fulda, Fulda, Germany; INSERM, US14 - Orphanet, Plateforme Maladies Rares, Paris, France; Campbell Family Mental Health Research Institute, Centre for Addiction and Mental Health, Toronto, ON, Canada; Institute of Medical and Molecular Genetics, Hospital Univ. La Paz, Madrid, Spain; University of Colorado Anschutz Medical Campus, Aurora, CO 80045, USA; Translational and Clinical Research Institute, Henry Wellcome Building, Framlington Place, Newcastle University, Newcastle-Upon-Tyne NE14LP, UK; Chinese HPO Consortium, Beijing, China; INSERM, US14 - Orphanet, Plateforme Maladies Rares, Paris, France; Department of Psychiatry, Brigham and Women's Hospital, Harvard Medical School, Boston, MA, USA; Center for Craniofacial and Dental Genetics, Department of Oral and Craniofacial Sciences, School of Dental Medicine, University of Pittsburgh, Pittsburgh, PA, USA; Center for Genomic Medicine, Parc Taulí Hospital Universitari, Institut d’Investigació i Innovació Parc Taulí (I3PT-CERCA), Sabadell, Spain; Center for Craniofacial and Dental Genetics, Department of Oral and Craniofacial Sciences, School of Dental Medicine, University of Pittsburgh, Pittsburgh, PA, USA; Department of Psychiatry, University of Michigan, Ann Arbor, MI, USA; University of Colorado Anschutz Medical Campus, Aurora, CO 80045, USA; Department of Biology and Medical Genetics, 2nd Medical Faculty of Charles University and University Hospital Motol, Prague, Czech Republic; Feinstein Institutes for Medical Research, Northwell Health, Manhasset, NY, USA; Discipline of Psychiatry & Mental Health, School of Clinical Medicine, Faculty of Medicine & Health, University of New South Wales, Sydney, NSW, Australia; Department of Biology and Medical Genetics, 2nd Medical Faculty of Charles University and University Hospital Motol, Prague, Czech Republic; Okinawa Prefectural Nanbu Medical Center & Children's Medical Center; School of Physical and Occupational Therapy, McGill University, Montreal, Quebec, Canada; Institute of Medical and Molecular Genetics, Hospital Univ. La Paz, Madrid, Spain; Dauten Family Center for Bipolar Treatment Innovation, Massachusetts General Hospital, Boston, MA, USA; Department of Biology and Medical Genetics, 2nd Medical Faculty of Charles University and University Hospital Motol, Prague, Czech Republic; Stark Neurosciences Research Institute, Departments of Psychiatry and Medical and Molecular Genetics, Indiana University School of Medicine, Indianapolis, IN, USA; INGEM/ToMMo, Tohoku University, Sendai, Japan; Data Collaboration Center, Data Science, Critical Path Institute, Tucson, AZ, USA; Department of Psychiatry, University of Toronto, Toronto, ON, Canada; Centro de Investigaciones en Anomalías Congénitas y Enfermedades Raras (CIACER), Universidad Icesi, Cali, Colombia; Molecular (epi) genetics lab, Bioaraba Health Research Institute, Araba University Hospital, Vitoria-Gasteiz, Spain; Department of Psychiatry, Massachusetts General Hospital, Boston, MA, USA; University of Colorado Anschutz Medical Campus, Aurora, CO 80045, USA; Department of Pediatrics, Dr. von Hauner Children's Hospital, University Hospital, LMU Munich, German center for Lung research (DZL), Munich, Germany; INSERM, US14 - Orphanet, Plateforme Maladies Rares, Paris, France; Division of Environmental Genomics and Systems Biology, Lawrence Berkeley National Laboratory, Berkeley, CA 94720, USA; The Jackson Laboratory for Genomic Medicine, Farmington, CT, USA; National Heart & Lung Institute & MRC London Institute of Medical Sciences, Imperial College London, London W12 0HS, UK; SNOMED International, London W2 6BD, UK; Department of Paediatrics, Institute of Developmental and Regenerative Medicine, University of Oxford, Oxford, UK; Universitätsklinikum Carl Gustav Carus, Medizinische Fakultät, TU, Dresden, Germany; Institute of Psychiatric Phenomics and Genomics (IPPG), LMU University Hospital, LMU Munich, Munich, Germany; Department of Psychiatry and Behavioral Sciences, SUNY Upstate Medical University, Syracuse, NY, USA; Department of Biology and Medical Genetics, 2nd Medical Faculty of Charles University and University Hospital Motol, Prague, Czech Republic; Department of Psychiatry, Dalhousie University, Halifax, NS, Canada; Exploratory Diagnostic Sciences, Berliner Institut für Gesundheitsforschung - Charité, Berlin, Germany; Department of Ophthalmology, Saarland University Medical Center UKS, Homburg/Saar, Germany; Chinese HPO Consortium, Beijing, China; National Institute of Allergy and Infectious Diseases, National Institutes of Health, Bethesda, MD 20892, USA; Eisenberg Family Depression Center, University of Michigan, Ann Arbor, MI, USA; Department of Psychiatry and Psychology, Mayo Clinic, Rochester, MN, USA; William Harvey Research Institute, Queen Mary University of London, London, UK; The Jackson Laboratory, Bar Harbor, ME, USA; Human Genetics Institute of New Jersey, Rutgers University, Piscataway, NJ, USA; Department of Psychiatry, University of Michigan, Ann Arbor, MI, USA; The National Alliance on Mental Illness, Arlington, VA, USA; European Bioinformatics Institute (EMBL-EBI), Wellcome Genome Campus, Hinxton CB10 1SD, UK; Exploratory Diagnostic Sciences, Berliner Institut für Gesundheitsforschung - Charité, Berlin, Germany; Department of Psychological Medicine, Institute of Psychiatry, Psychology & Neuroscience, King's College London, London, UK; The Jackson Laboratory for Genomic Medicine, Farmington, CT, USA; Institute for Research and Health Policy Studies, Tufts Medicine, Boston, MA 2111, USA; Institute of Medical and Molecular Genetics, Hospital Univ. La Paz, Madrid, Spain; Department of Biology and Medical Genetics, 2nd Medical Faculty of Charles University and University Hospital Motol, Prague, Czech Republic; Translational and Clinical Research Institute, Henry Wellcome Building, Framlington Place, Newcastle University, Newcastle-Upon-Tyne NE14LP, UK; Neurodevelopmental and Behavioral Phenotyping Service, National Institute of Mental Health, Bethesda, MD, USA; Department of Biology and Medical Genetics, 2nd Medical Faculty of Charles University and University Hospital Motol, Prague, Czech Republic; Department of Genetics, University Medical Center Groningen, Groningen, Netherlands; Critical Path Institute, Tucson, AZ, USA; Department of Biology and Medical Genetics, 2nd Medical Faculty of Charles University and University Hospital Motol, Prague, Czech Republic; Department of Biomedical Informatics, University of Colorado Anschutz Medical Campus, Aurora, CO 80045, USA; Chinese HPO Consortium, Beijing, China; Department of Obstetrics and Gynecology, Columbia University Irving Medical Center, New York, NY, USA; National Heart & Lung Institute & MRC London Institute of Medical Sciences, Imperial College London, London W12 0HS, UK; Rare Disease Ghana Initiative, Accra, Ghana; Rare Disease Ghana Initiative, Accra, Ghana; National Center on Birth Defects and Developmental Disabilities, Centers for Disease Control and Prevention, Atlanta, GA, USA; Institute for Research and Health Policy Studies, Tufts Medicine, Boston, MA 2111, USA; Chinese HPO Consortium, Beijing, China; Centre for Regenerative Medicine, Institute for Regeneration and Repair, Institute for Stem Cell Research, University of Edinburgh, Edinburgh, UK; Chinese HPO Consortium, Beijing, China; Department of Psychological Medicine, Institute of Psychiatry, Psychology & Neuroscience, King's College London, London, UK; Database Center for Life Science, Joint Support-Center for Data Science Research, Research Organization of Information and Systems, Japan; Department of Psychiatry, University of British Columbia, Vancouver, BC, Canada; Department of Psychiatry, University of Michigan, Ann Arbor, MI, USA; Psychological Medicine, Institute of Psychiatry, Psychology and Neuroscience, King's College London & South London and Maudsley NHS Foundation Trust, Bethlem Royal Hospital, Monks Orchard Road, Beckenham, Kent, London SE5 8AF, UK; Department of Human Genetics, Bioscientia Healthcare GmbH, Ingelheim, Germany; Department of Psychiatry and Behavioral Sciences, Johns Hopkins University School of Medicine, Baltimore, MD 21287, USA; Faculty of Medicine and Health, The University of Sydney, Camperdown, Australia; Institute of Human Genetics, Pontificia Universidad Javeriana, Bogotá, Colombia; Institute of Health Information and Statistics of the Czech Republic, Prague, Czech Republic; University of Colorado Anschutz Medical Campus, Aurora, CO 80045, USA; The Jackson Laboratory for Genomic Medicine, Farmington, CT, USA; Division of Environmental Genomics and Systems Biology, Lawrence Berkeley National Laboratory, Berkeley, CA 94720, USA; Department of Biomedical Informatics, University of Colorado Anschutz Medical Campus, Aurora, CO 80045, USA; The Jackson Laboratory for Genomic Medicine, Farmington, CT, USA; Division of Environmental Genomics and Systems Biology, Lawrence Berkeley National Laboratory, Berkeley, CA 94720, USA; Ada Health GmbH, Berlin, Germany; University of Colorado Anschutz Medical Campus, Aurora, CO 80045, USA; The Jackson Laboratory for Genomic Medicine, Farmington, CT, USA

## Abstract

The Human Phenotype Ontology (HPO) is a widely used resource that comprehensively organizes and defines the phenotypic features of human disease, enabling computational inference and supporting genomic and phenotypic analyses through semantic similarity and machine learning algorithms. The HPO has widespread applications in clinical diagnostics and translational research, including genomic diagnostics, gene-disease discovery, and cohort analytics. In recent years, groups around the world have developed translations of the HPO from English to other languages, and the HPO browser has been internationalized, allowing users to view HPO term labels and in many cases synonyms and definitions in ten languages in addition to English. Since our last report, a total of 2239 new HPO terms and 49235 new HPO annotations were developed, many in collaboration with external groups in the fields of psychiatry, arthrogryposis, immunology and cardiology. The Medical Action Ontology (MAxO) is a new effort to model treatments and other measures taken for clinical management. Finally, the HPO consortium is contributing to efforts to integrate the HPO and the GA4GH Phenopacket Schema into electronic health records (EHRs) with the goal of more standardized and computable integration of rare disease data in EHRs.

## Graphical Abstract

**Graphical Abstract:**
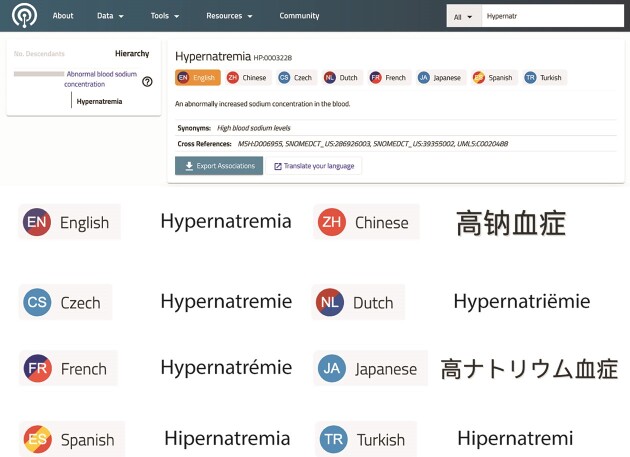

## Introduction

The Human Phenotype Ontology (HPO) is a rich representation of the diversity of human phenotypes; it is a globally recognized standard for the computational encoding of ‘deep phenotype’ data. Because it is logically structured, the HPO enables computational inference and sophisticated algorithms that support combined genomic and phenotypic analysis in a range of applications spanning basic research to clinical care. The HPO is a flagship component of the Monarch Initiative (1), comprising resources and community efforts to enable interoperability across data sources and organisms. Examples of HPO applications include genomic interpretation for diagnostics, gene-disease discovery, machine learning (ML) and electronic health record (EHR) cohort analytics – all of which assist in realizing the promise of precision medicine. Additionally, the HPO provides a comprehensive corpus of phenotype annotations (HPOA) corresponding to each of over 8100 rare diseases. HPO has become a global standard for use in rare disease diagnostic tools: almost all clinical genetics diagnostic tools, including Exomiser, LIRICAL, SimulConsult, PhenoTips and Face2Gene, now leverage the HPO to encode and compute over patient features in the context of genomic variant classification. Further, by enabling data exchange, HPO unites an ever-growing community of users and contributors across the world, illuminating the natural history of disease, revealing new diseases, supporting patient registries and n-of-1 matchmaking for diagnosis (2) and aiding numerous national genomic initiatives and biobanking programs (3). In this update to previous articles in the Nucleic Acids Research database issue (4–7), we discuss recent additions to the project, including translations to 10 languages, new HPO terms and annotations for a number of medical fields, and efforts to integrate HPO with electronic health records (EHRs).

## The Human Phenotype Ontology internationalization effort

The HPO Internationalization Effort (HPOIE) is a loose alliance of translation communities to coordinate efforts. Our vision is to break down language barriers and ontology curation internationally. The HPOIE is loosely divided into a Coordination Committee and language-specific working groups (LSWG). The Coordination Committee is run by members of the HPO Operations Committee (SK and NM at the time of writing) and is concerned with onboarding new LSWGs and pushing updates to HPO to supported curation platforms. The LSWGs are concerned with managing translations into a particular language and coordinating disparate efforts that involve translations into the same language. A list of current translation efforts can be found in Table 1.

**Table 1.** Languages that are officially part of the HPO Internationalization Effort

Language	Labels	Definitions	Updated in 2023
Chinese	16 691	0	Yes
Czech	14 362	11 623	Yes
Dutch	12 448	38	−
Dusun	13	0	−
French	14 220	473	Yes
Japanese	17 649	0	Yes
Nyangumarta	201	0	−
Spanish	17875	0	Yes
Tiwi	83	0	−
Turkish	13 888	11 086	−

Dusun, Tiwi and Nyangumarta profiles reflect only the subsets of HPO that are particularly relevant to their communities. All other efforts were designed to be comprehensive, but only Czech, Chinese, French, Japanese and Spanish were updated in 2023.

The internationalization effort involves fostering collaboration between different groups translating the same language by harmonizing and choosing the ‘best’ words in different countries and continents that use the same language (e.g. Spanish in Spain and Latin American countries). Additionally, we have standardized the representation of the translation profiles, capturing rich metadata that can be used by downstream sources to separate, for example, professional manual translations from automated ones. We have set up a software framework to coordinate the release of language profiles with HPO releases.

We plan to include additional languages as well as layperson translations (8) in the HPOIE as translation efforts progress and invite interested groups to contact us.

### International edition and language profiles

Seven comprehensive translations of the HPO are available in addition to three partial translations (Table 1). Since April 2023, we have been publishing a special release version of the HPO ontology, the HPO International Edition (HPI), which includes translations for Turkish, Czech, French and Dutch. Language profiles are the translated subsets of HPO that come with metadata such as ‘translation status’ and ‘translation date’. They are curated in a simple tabular format, Babelon TSV. Babelon is a data model for language profiles maintained using LinkML (https://linkml.io/), which provides a standard translation from Babelon TSV files into OWL. Language profiles are versioned alongside the primary releases of the HPO in four forms: a special HPO international edition that corresponds to the standard HPO release including all the language profiles (hp-international.owl); a language specific version of the HPO (e.g. hp-fr.owl for the French language version of the HPO in OWL format); a set of tabular files that correspond to the translations of the labels and definitions (e.g. hp-fr.babelon.tsv for the French translations) and a special file that contains language-specific synonyms (e.g. hp-fr.synonyms.tsv). While labels and definitions are translated directly, we do not attempt to translate each synonym directly. Instead, a list of synonyms appropriate to each language is curated separately.

As labels and definitions undergo change, some of the translations may need to be revised. For each translated term, we record a ‘translation status’ that can be the following: ‘candidate’ (translation has been suggested by an entity [algorithm, person] outside the core team managing the translation), ‘under review’ (translation has been suggested by an entity [algorithm, person] inside the core team managing the translation, but not yet officially ratified by the LSWG) and ‘official’ (translation has been accepted by the core team managing the language profile).

When a label changes, we do not discard the translation of the corresponding term, but instead deprecate its status from OFFICIAL to CANDIDATE, meaning that it needs to be re-curated by the translation team. This status change happens automatically with each release, and an accompanying report (e.g. updated-labels-to-candidate-status-fr.tsv) is made available for the LSWG.

### Recent accomplishments in HPO translation

All HPO translations are available on the HPO GitHub repository.

The **Japanese translations** were developed by the GEnome Medical alliance (GEM) Japan Clinical Phenotype group for the purpose of precise phenotyping of patients in genome projects. Expert translation by clinical geneticists and AI translation were utilized. This translation has been used widely in clinical genome projects for rare diseases in Japan.

The translation of HPO terms from English to **Turkish** commenced as a personal endeavor by Zafer Yüksel, with the goal of standardizing patient phenotype information in laboratory and hospital information management systems using HPO terms and supporting computational tools for differential diagnostic support based on patient notes. These tools are also designed to automate the interpretation of genetic data for the accurate diagnosis of genetic disorders. The overarching aim is to make this translation initiative accessible to users in Turkey under the guidance of institutional leadership, while also inviting their valuable contributions.

The **Chinese translation** of HPO was maintained and constantly updated by the Chinese HPO (CHPO) consortium. CHPO was established in 2016 as an open platform to synergize the efforts on research on rare diseases. Under the umbrella of CHPO, experts from different clinical domains in China provided translation of HPO terms to the corresponding Chinese terms.

The **Dutch translation** for the HPO was created by the Human Genetics department of the Radboud University Medical Center to store phenotypic data using HPO identifiers extracted from Dutch language clinical notes.

The three **partial translations of Indigenous languages (Dusun, Nyangumarta, Tiwi)** were created by Lyfe Languages champions in consultation with and the approval of Community Elders. The Lyfe Languages project is aimed at breaking down barriers to communication by translating medical resources and terminology into the languages spoken by Indigenous communities. The translation was used to prepare and publish health and educational materials initially for rare diseases, then for COVID-19 and subsequently extending to all health domains.

The **French translations** of the HPO, initiated by the Paris Orphanet team, will be integrated as a phenotypic descriptor in the French National Rare Disease Registry (BNDMR) a national epidemiological and public health tool funded by the French Ministry for Solidarity and Health. The BNDMR gathers data for all patients treated in centers qualified for their expertise in rare diseases in France. The HPO French translation is also being integrated in the new RDK (Rare Disease Knowledge) French web and mobile application for healthcare professionals, which offers diagnostic guidance to identify patients affected by a rare disease.

The **Spanish translation** of the HPO terminology was created in 2013 by a group of Spanish researchers. Soon after, a team of Colombian investigators was added to facilitate the use of Spanish in Spanish-speaking Latin American countries. The Spanish HPO translation is used by many institutions and hospitals and currently is the gold standard terminology applied to share information for national research projects such as the IMPACT initiative, the ENOD program and the Spanish Network for Research on Rare Diseases (CIBERER). The HPO codification has been introduced in the vast majority of Spanish departments of Clinical Genetics as the chosen ontology for phenotypic description of patients. Currently, the Spanish translations are maintained and curated by a multidisciplinary team of researchers (HPO-Spanish Initiative) led by CIBERER.

The **Czech translation** was started by a team of clinical geneticists from the Department of Biology and Medical Genetics of 2nd Medical Faculty of Charles University and University Hospital Motol, which was joined by the National Center for Medical Nomenclatures and Classifications (NCMNK) of the Institute of Health Information and Statistics of the Czech Republic. The English version is already used in the Czech Republic in variant interpretation software and in bioinformatic analyses and pipelines. The intent of the translation effort is better adoption of HPO by Czech healthcare professionals and its implementation in health information and laboratory information management systems and registries. Localized versions could also be used for text mining from medical reports which are only available in the Czech language. Some draft translations were created with the help of AI translation tools, but all final translations were curated and approved by a human expert.

### The HPO international edition

**Figure 1.**
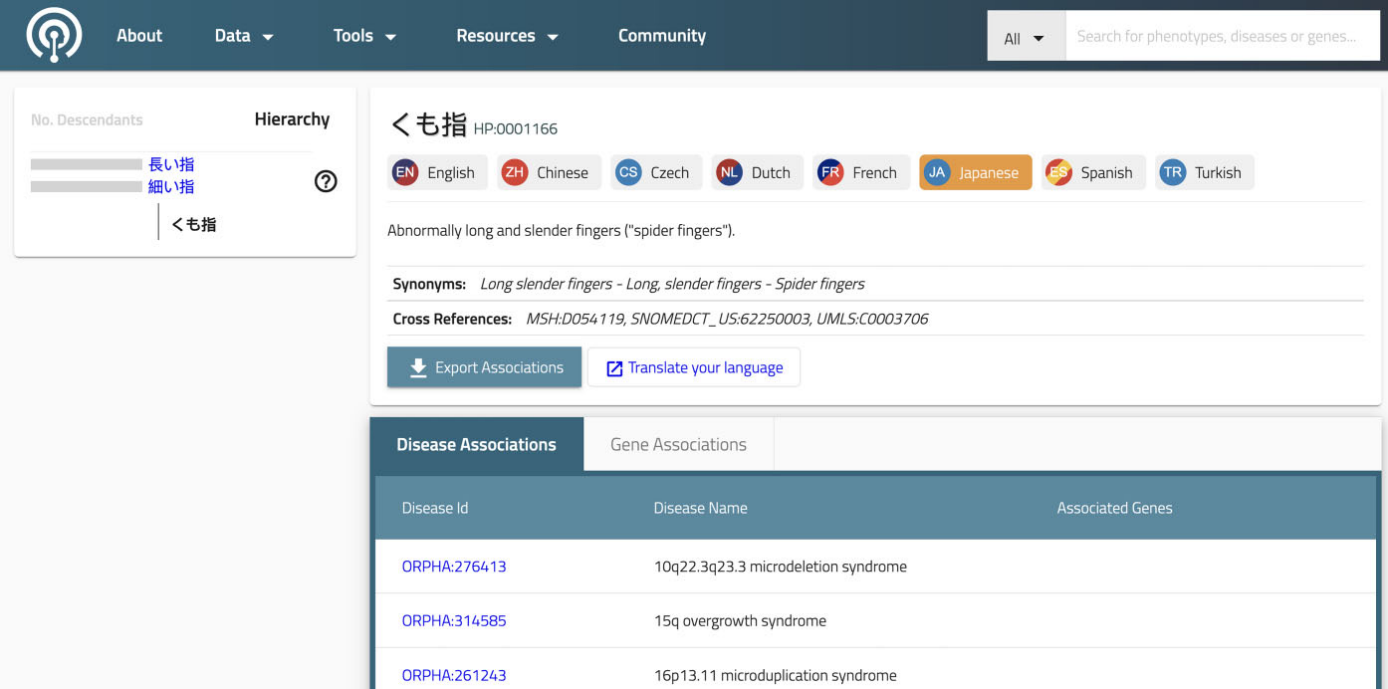
Screenshot of internationalized HPO Web application. For each term, users can choose from the available languages (seven, in this example) in addition to English. Term labels, definitions and synonyms are shown in the chosen language if translations are available; otherwise, the English version is shown.

In our most recent version of the HPO web app, we have integrated support for translations (Figure 1). Currently we offer a way to translate both terms and the class hierarchy on the left, if the respective translation exists.

Services such as the widely used Ontology Lookup Service (9) have integrated HPO translations and allow toggling translations on and off when browsing the ontology. The Ontology Access Kit (OAK) has implemented multi-language support in response to our work, which allows querying HPO with a ‘preferred-language’ parameter.

## Updates on annotations and ontology content

### Updates to the ontology

Since our last NAR update (HPO, October 2020 release, (5)), we have added 2239 terms (Table 2). We will describe some of the major efforts involved in the next section. In addition to adding new terms, we have added or updated 612 definitions to existing terms, added 1377 new synonyms and renamed 671 classes (these stats were generated using OAK diff). Collaborative engagement can be measured by interactions with our issue tracker and GitHub repository. Since 15 September 2020 (the cut-off date of the last update), we have received contributions from 177 individuals, which opened a total of 3715 issues and made a total of 5159 comments on issues.

**Table 2.** Distribution of the 2239 new terms added to the HPO since the October 2020 release. Counts are shown for each of the main branches

**Branch**	**Label**	**New terms**
HP:0032443	Past medical history	58
HP:0001574	Abnormality of the integument	105
HP:0000152	Abnormality of head or neck	82
HP:0012823	Clinical modifier	85
HP:0001939	Abnormality of metabolism/homeostasis	354
HP:0000005	Mode of inheritance	13
HP:0001871	Abnormality of blood and blood-forming tissues	62
HP:0001507	Growth abnormality	2
HP:0002664	Neoplasm	60
HP:0001197	Abnormality of prenatal development or birth	99
HP:0000478	Abnormality of the eye	28
HP:0001626	Abnormality of the cardiovascular system	154
HP:0033127	Abnormality of the musculoskeletal system	133
HP:0002715	Abnormality of the immune system	307
HP:0025142	Constitutional symptom	18
HP:0002086	Abnormality of the respiratory system	154
HP:0040064	Abnormality of limbs	43
HP:0000119	Abnormality of the genitourinary system	408
HP:0000707	Abnormality of the nervous system	339
HP:0025354	Abnormal cellular phenotype	13
HP:0000818	Abnormality of the endocrine system	39
HP:0045027	Abnormality of the thoracic cavity	2
HP:0000598	Abnormality of the ear	4
HP:0025031	Abnormality of the digestive system	98

Because of multiple inheritance, the counts do not add up to 2239.

**British Spelling synonyms**. The HPO uses American spelling conventions (e.g. ‘Palpebral edema’ [HP:0100540]). Where applicable, British spellings are automatically provided as synonyms (e.g. ‘Palpebral oedema’) labeled with the hp:uk_spelling tag. A total of 1020 British synonyms were available in the September 2023 release.

### Updates to the annotations

The HPO annotation files have been versioned alongside HPO since February 2022. Since our previous publication in 2020 we have added about 49 000 new annotations. The annotations now include citations to a total of 9573 PubMed identifiers (PMIDs).

### Improved coverage of specific clinical areas

The HPO team has hosted regular workshops with clinical experts since the very beginning of the project in 2008. These workshops extend the resource by adding new terms and revising existing terms by adding or improving definitions, synonyms, or the structure of the ontology. Additionally, disease annotations are added. Most workshops involve discussions among domain experts and the HPO team on the ontological modeling of clinically relevant phenotypic abnormalities. Below, we summarize recent efforts to improve the HPO’s coverage of specific clinical areas.

**Behavior**. Behavioral disorders alone account for a substantial proportion of human disease burden (10) and many diseases involve significant behavioral components and comorbidities. These include conditions that affect the core mood and anxiety states, perceptions and experiences related to interactions with the world, and behaviors motivated by internal states. However, the subjective and contextual nature of behavioral abnormalities creates challenges for representation as ontology terms (11). Furthermore, we need a way to represent changes in behavioral milestones (such as learning to walk). To address these challenges, we conducted two workshops in 2023 to create new behavioral terms and improve the structure of the behavioral portion of the HPO. The first workshop, held at the National Institute of Mental Health (NIMH), focused on phenotypic features seen in neurodevelopmental disorders such as autism spectrum disorder. This two-day workshop included 20 participants with expertise in psychology, pediatrics and data science. The second workshop took place in Chicago prior to the annual meeting of the International Society for Bipolar Disorders. This meeting involved a day dedicated to creating behavioral terms to describe mood disorders. The workshop included an international collaboration of 40 participants with primary expertise in bipolar disorder. Efforts in this field are ongoing and we welcome new collaborators.

**Prenatal medicine**. Recently, emphasis has been placed on expanding the ontology to better capture prenatal phenotypes, especially phenotypes that may change depending on gestational age (12). To address the previous limitations of HPO in representing prenatal phenotypes, a series of workshops was initiated involving experts in prenatal medicine and genetics. The International Fetal Genomics Consortium uses the updated HPO and Phenopackets to aggregate and compute large amounts of prenatal genomic and phenotype data in a cloud-based repository for research and clinical use.

**Inborn errors of immunity** (IEI) comprise a large group of disorders, each characterized by a specific dysfunction of the immune system. More than 485 different IEIs have been described with a wide variety of clinical and immunological phenotypes ranging from increased susceptibility to infections to immune dysregulation, presenting as autoimmunity, hyperinflammation, autoinflammation, lymphoproliferation or malignancy. Specific IEIs display distinct clinical features as well as characteristic laboratory phenotypes (13). Clinical experts in IEI agree that a major barrier to the adoption of the HPO terminology is the lack of disease-specific HPO terms to accurately describe patients with IEI and their corresponding immune profiles (14). That is why in 2018, a collaborative initiative between ERN-RITA (European Network on Rare Primary Immunodeficiency, Autoinflammatory and Autoimmune Diseases), ESID (European Society for Immunodeficiencies) and ISSAID (International Society of Systemic Autoinflammatory Diseases) was launched to improve HPO terminology for IEIs by coupling established bioinformatic methodologies with expert review. To date this effort has resulted in the creation of over 60 new HPO terms, restructuring of over 200 terms and re-annotation of 109 IEIs. Although the HPO for IEIs is only partly curated, the consortium's ongoing project has already significantly expanded the description and categorization of phenotypic similarities of disorders; when these data are then combined with the available filtering algorithms of potential disease-causing genetic variants, the likelihood of matching patients to the correct diagnosis is substantially improved (15,16).

**Orofacial clefts**. Orofacial clefts (OFCs) are a heterogeneous group of disorders including clefts of the lip (CL), the palate (CP) or both (CLP) (17). The OFC phenotypes are reported in terms of laterality (unilateral or bilateral), side (left or right) and completeness (complete or incomplete) of the CL and/or CP. All laterality/side/completeness permutations have been observed in affected individuals, but representing all of them by individual HPO terms would be too onerous. We created a data model inspired by Phenopackets that allows the use of the HPO modifiers ‘Left’ (HP:0012835) and ‘Right’ (HP:0012834) to specify the side of the phenotypic anomaly. We reviewed the orofacial cleft branch of HPO (HP:0000202), and created new terms representing the general observed phenotype, e.g. ‘Asymmetric bilateral cleft lip’ (HP:5201014), representing a bilateral CL in which the CL is complete on one side and incomplete on the other side. More phenotypic details can be recorded using the left/right modifiers in association with generic terms reporting the completeness on the cleft, e.g. ‘Complete cleft of the upper lip’ (HP:5201009). Other terms are under development to capture subtle defects such as microforms, and to update definitions of all related HPO terms.

**Epileptology**. Sudden unexpected death in epilepsy (SUDEP), an unexpected non-traumatic and non-drowning death in an otherwise healthy individual with epilepsy, has been an important addition to the HPO (HP:0033258) (18). The precise mechanisms leading to SUDEP remain elusive, but are thought to involve cardiac arrhythmias, respiratory insufficiency and dysfunction of arousal. The risk of SUDEP is known to be elevated in some monogenetic epilepsies, including Dravet syndrome (19), and it was prioritized for research by patients, their advocates and healthcare professionals. The representation of SUDEP in the HPO will facilitate research into the genetic underpinnings of this rare but severe condition that is methodologically difficult to address: its association with deletion of 1p36 was suggested in our recent largest ever phenome-wide association study of epilepsies (20). Hippocampal sclerosis, a common cause of drug-resistant epilepsy, characterized by a specific pattern of cell loss and gliosis in the hippocampus, can now be annotated specifically with a new HPO term (HP:0033258) that captures both pathological and imaging features (21). Electroencephalography (EEG) is a crucial tool in the diagnosis and classification of epilepsy, and despite proposed standardization, harmonization of clinical EEG reports remains a challenge (22). The process of harmonizing reported EEG phenotypes has emerged as a new focus to allow researchers and clinicians to communicate more effectively to discover the genetic factors associated with specific EEG patterns (23).

**Male infertility genetics**. Recently, the HPO has introduced multiple new terms that are related to male infertility. These terms branch from the term ‘decreased fertility in males’ (HP:0012041) and comprise a spectrum of phenotypes that pertain to sperm count, testicular histology phenotypes, aberrant sperm morphology (teratozoospermia) and sperm motility (asthenozoospermia) (24). The terms are partly based on the phenotypes described in the WHO laboratory manual for the examination and processing of human semen (25). The improved male infertility HPO terms will facilitate deciphering the genetic reasons for male infertility, which may help to predict the chances of successful testicular sperm retrieval in azoospermic males (26).

**Autoantibodies**. Recent technological advances have improved the diagnosis of autoimmune diseases, notably through the identification of new autoantibodies. In the past, there was no publicly available clinical database to capture the presence of autoantibodies in medical records in a standardized way. Indeed, in 2020, only 48 autoantibodies were present in the HPO database. Multi-institution work carried out with the French rare disease health networks and the ERN ERKNet has made it possible to add more than 200 new antibodies recorded as HPO biological signs, in the form ‘Anti-X antibody positivity’. Synonyms and full names have been added after curation of the literature when available. All new terms generated were classified under the group ‘Autoimmune antibody positivity’ (HP:0030057).

**Arthrogryposis multiplex congenita (AMC)**. AMC is characterized by multiple congenital contractures, encompassing many different conditions and involving over 400 genes (27,28). A registry for AMC funded by Shriners’ Children combines a phenotypic description and genotyping using whole genome sequencing (29). To provide a standardized language for AMC, common data elements (CDEs) have been identified by an international consortium of 45 experts in orthopedics, genetics, neurology, rehabilitation, obstetrics and lived experience across 11 countries. The AMC-related phenotypic terms were mapped to the HPO using two HPO branches: ‘Abnormality of the musculoskeletal system’ (HP:0033127) and ‘Abnormality of the limbs’ (HP:0040064). Extensive collaborations with the HPO team resulted in data curations for 27 new HPO terms, 38 restructurings and 20 re-annotations with significant improvements for describing contractures in AMC (30).

**Cerebral palsy (CP)** is a common neurodevelopmental disorder affecting around 1 in 500 children, characterized by permanent but evolving disorders of movement and posture due to non-progressive disturbances in the developing fetal or infant brain. The heterogeneity and complex genetic etiologies of CP have hindered progress in understanding its molecular causes, and diagnosis remains primarily clinical, lacking specific diagnostic tests (31). Shriners Children's is spearheading research to establish whole genome sequencing for 500 children with CP, including existing classifications, functional descriptors and magnetic resonance imaging data, applied to the CP cohort. Utilizing the HPO as a framework, this project will link genomic and phenomic data, contributing to the advancement of genetic precision medicine in CP.

**Pulmonology**. Diffuse alveolar hemorrhage (DAH) in children is a rare condition resulting from numerous different underlying diseases. A series of virtual workshops were held to add new terms, revise definitions of existing terms and improve the hierarchy of the HPO with a focus on DAH and other pediatric diseases of the lung. HPO terms and disease annotations were created as needed to cover phenotypic characteristics of patients within the chILD-EU register (European international management platform for children's interstitial lung disease) and from the specialized literature. An evaluation of the application of HPO to support differential diagnosis in DAH was recently presented (32). Additionally, to link the HPO terms to diseases a large number of new diagnoses from the diagnostic catalog of the chILD-EU register were introduced into the Mondo Disease Ontology.

**Phenotypic features of COVID-19 and long COVID**. The COVID-19 pandemic highlighted the importance of genomic surveillance for public health intervention and decision making. A key factor was to better allow researchers to identify phenotypic information associated with the disease through sequence-associated contextual information that described clinical case descriptions, including but not limited to disease-associated signs and symptoms. Coronavirus disease 2019 (COVID-19) affects diverse organ systems, including the lungs, digestive tract, kidneys, heart and brain and is caused by infection with Severe Acute Respiratory Syndrome Coronavirus-2 (SARS-CoV-2). Post-acute sequelae of SARS-CoV-2 (colloquially referred to as Long COVID) is an often debilitating illness that occurs in at least 10% of SARS-CoV-2 infections. A computational standard for describing the clinical manifestations of long COVID has been lacking. The HPO team reviewed 59 manuscripts that described clinical manifestations in 81 cohorts three weeks or more following acute COVID-19, and mapped 287 unique clinical findings to HPO terms. HPO helped address the gaps in this vocabulary by accepting and curating new terms included but not limited to those describing circulatory abnormalities such as ‘Silent Hypoxemia’, inflammatory skin abnormalities such as ‘Pseudo-chilblains on toes’ (i.e. COVID toes), and skin abnormalities describing various ‘Cyanosis’ phenotypes (33). These terms have since been used to describe data via the Public Health Alliance for Genomic Epidemiology (PHA4GE) SARS-CoV-2 contextual data specification, the Canadian COVID Genomics Network (CanCOGeN) SARS-CoV-2 contextual data specification (24), and have been used for clustering patients within the National Covid Cohort Collaborative (N3C) to aid in mechanism discovery and identify treatments (34,35). Additionally, layperson synonyms and definitions were created that can be used to link patient self-report questionnaires to standard medical terminology (33).

**Inheritance**. The Gene Curation Coalition (GenCC) is a global effort to harmonize gene-level resources (36). As part of our effort to facilitate the harmonization of gene-disease validity assessments and support robust variant classification within the ACMG/AMP framework, we have proposed a standardized terminology to describe inheritance and allelic requirement (37). Most GenCC member group terms for inheritance mapped to existing HPO terms such as ‘Autosomal dominant inheritance’ (HP:0000006), but few groups had dedicated terms for allelic requirement. Qualifier terms were used by some groups to add further detail but there was limited consistency between groups for these. We formed a working group to restructure and streamline HPO inheritance terms. Three novel subdivisions within mode of Inheritance were introduced, to distinguish Mendelian inheritance, non-Mendelian inheritance and qualifiers such as ‘Genetic anticipation’ (HP:0003743). New inheritance terms were added where needed and allelic balance terms were derived from first principles. Finalized terms for inheritance and allelic requirement, which had been derived independently of each other, were aligned such that each mode of inheritance has a synonymous allelic requirement term to describe the context necessary to cause disease.

**SimulConsult curation**. The HPO terminology is being expanded using the findings identified in the literature by clinicians curating information for the SimulConsult diagnostic decision support software. Of the 5043 non-gene findings currently in SimulConsult, 1929 were identified in 2022 as not having an HPO term and 186 identified among terms added to the diagnostic software since then. For these 2115 terms, to date 532 new HPO terms have been added, and it is planned to add the bulk of the others. Predominant among these terms have been laboratory findings in genetic diseases not previously emphasized in HPO, and clinical and laboratory findings in non-genetic diseases, which the diagnostic software has expanded to cover. These findings have been identified by clinician curators in the literature as being useful for diagnosis, thereby supporting the mission of HPO to provide the infrastructure for making medicine computable.

### Interoperability with other ontologies

**The ontology of biological attributes (OBA)**. OBA provides a formalized, species-independent, interoperable and standardized representational framework for observable attributes that are characteristics of biological entities, organisms, or parts of organisms (38). In contrast to HPO and other existing phenotype ontologies, the OBA attribute and phenotypic trait categories are defined in such a way that the definitions are indifferent of any abnormality that may manifest in relation to a wild-type or any other reference. For example, genome-wide association studies (GWAS) using the OBA term OBA:2045209 ‘level of taurine in blood serum’ for their genetic data annotation are related to different abnormal phenotypes like ‘Hypotaurinemia’ (HP:0500182) or ‘Hypertaurinemia’ (HP:0500181). The same logical patterns, based on the Entity-Quality model (39), are used to define attributes in OBA as in HPO. This allows not only seamless classification of phenotypic abnormalities (HPO) under attributes (OBA), but specifically the integration of human and model organism phenotypes with GWAS, Quantitative Trait Loci (QTL) mappings or any or any other population-focussed measurable trait data.

**Aligning the Human Phenotype and Mammalian Phenotype Ontologies**. To foster the use of model organisms for human health research, we are mapping Human and Mammalian (40) Phenotype ontology terms using the Simple Standard for Sharing Ontological Mappings (SSSOM)-based method (41) to review and refine the alignment of these ontologies. During this mapping, discrepancies between the definitions, equivalence axioms and placement of terms were identified, discussed and resolved whenever possible. These updates improve the reliability of these ontologies in automated tools and increase the probability that terms that share similar labels also share similar definitions. To date, 1343 mappings have been created.

**HPO-SNOMED mapping**. The integration of HPO with SNOMED CT (Systematised Nomenclature of Medicine-Clinical Terms) is an important step in the harmonization of medical terminologies (42). This mapping aims to bridge the gap between the hierarchical structure of HPO terms and the widely used clinical vocabulary of SNOMED. The HPO-SNOMED mapping improves semantic interoperability and facilitates the translation of complex phenotypic data into actionable clinical information. This collaborative project serves as a cornerstone for standardizing phenotype descriptions across different healthcare systems and research databases.

**Biomappings**. The Biomappings workflow was used to generate lexical mappings from HPO to MeSH and other widely used ontologies. These were manually curated, resulting in nearly 300 mappings readily available in the SSSOM format (43).

**The Medical Action Ontology**. The Medical Action Ontology (MAxO) is designed to organize medical procedures, therapies and interventions in a structured way. Currently, MAxO contains 1757 terms describing activities and measures undertaken as a part of clinical management that collectively we refer to as medical actions. In addition to pharmaceutical treatment, medical actions include surgical procedures, ablations, treatment with biologics, behavioral and cognitive interventions, deep brain stimulation and many others. We have created a database of over 16 000 annotations that describe diagnostic modalities for specific phenotypic abnormalities as defined by the HPO. Additionally, 413 annotations are provided for medical actions for 189 rare diseases. The development of MAxO is closely coupled to the Mondo Disease Ontology (1) and HPO and expands the scope of our computational modeling of diseases and phenotypic features to include diagnostics and therapeutic actions.

## Applications and use cases

### *Fenominal*: a tool for HPO concept recognition

The process of deep phenotyping can be slow, tedious and error-prone if performed manually, which can be a substantial barrier to uptake from clinicians. In recent years, however, natural language processing (NLP) tools have been developed and have replaced, to a certain extent, the need for manual encoding of phenotype profiles. Within the landscape of such tools, *fenominal*(*"****F****abulous ph****ENO****type****MIN****ing****AL****gorithm*) is a ‘phenomenal’ Java software library developed by the Monarch team to perform HPO concept recognition via a command-line interface or a user-friendly graphical user interface. A number of approaches have been developed to address the challenges governing the phenotype CR task, such as ambiguity, use of metaphorical expressions (e.g. ‘Angel-shaped phalanx’ [HP:0032078]), negation and complex or nested structures. To increase adoption of HPO and the use of fenominal for EHRs, we focused on a challenge largely ignored or assumed to be implicitly solved by the existing phenotype CR tools: typographical errors often encountered in real-world clinical settings. Typographical errors can be seen as a subclass of lexical variation in the context of the concept recognition task. They are significantly less structured than standard lexical variation, with the semantics of the tokens being harder to predict and interpret. We addressed this challenge by developing a method in fenominal inspired by the BLAST algorithm for biosequence alignment (44) that screens texts for potential matches on the basis of matching k-mer counts and scores candidates based on conformance to typical patterns of spelling errors derived from 2.9 million clinical notes. We produced a gold standard corpus for typographical errors and built a sequence alignment-inspired method to efficiently perform entity linkage. Validation of the method showed an increase of 10% in recall on scientific publications and 20% increase in recall on EHR records, hence supporting a significant enhancement of the entity linking task.

### Software that supports clinical diagnostics

A growing number of diagnostic products use the HPO to encode precise diagnostic information.

**SimulConsult** uses an algorithm that combines a variety of patient data—including symptoms, family history and laboratory results—to generate a list of differential diagnoses. By integrating with the HPO, the tool increases the precision and individualization of the diagnostic process.

**PhenoTips** was originally developed to meet the needs of clinical geneticists and provides a platform for the collection and analysis of patient phenotypic data. The software uses the HPO to code phenotypic abnormalities in a standardized way, promoting consistent data entry and interpretation by different healthcare providers and researchers. This standardization is essential for collaborative studies, especially in the field of rare diseases where patient cohorts are small and widely dispersed.

**Face2Gene** uses machine learning algorithms for facial analysis to help doctors diagnose genetic diseases. It uses HPO to assign facial phenotypes to possible genetic causes. This integration of face recognition technology and phenotype ontology provides a multidimensional approach to diagnostic support that complements traditional diagnostic methods.

**SAMS** (Symptom Annotation Made Simple) is a web application that allows users to enter diagnoses (from Orphanet and OMIM) as well as the presence or explicit absence of clinical signs or symptoms from the HPO, including an unlimited number of modifying terms for each sign. SAMS is aimed at clinicians with little HPO experience and provides an intuitive interface. SAMS also can export and import GA4GH Phenopackets (45).

**SUOG** (Smart Ultrasound in Obstetrics and Gynecology) is a EU-funded decision support system for early pregnancy and fetal disorders based on semantic reasoning and ML. SUOG provides step by step guidance during prenatal scans and leverages a knowledge base with a dedicated ontology (46). The SUOG ontology (v 3.70i) contains fine-grained fetal ultrasound phenotype descriptions, of which 1358 are mapped under HPO classes or coded with HPO.

### GA4GH phenopacket schema

**Figure 2.**
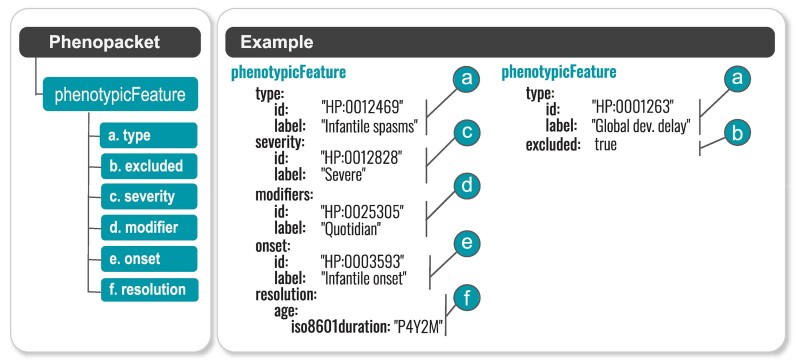
The Phenotypic Feature element of the GA4GH Phenopacket Schema. This element contextualizes HPO annotations by allowing the severity, onset, resolution, and other characteristics (modifiers) of the phenotypic observation to be specified. In this example, two phenotypic observations are given: (i) severe, daily (quotidian) infantile spasms with infantile onset (between month two and twelve of life) and resolution at the age of 4 years, 2 months and (ii) exclusion of global developmental delay (that is, this feature was assessed and explicitly found not to be present).

The Phenopacket Schema is a Global Alliance for Genomics and Health (GA4GH) standard for sharing disease and phenotype information that characterizes an individual person, linking that individual to detailed phenotypic descriptions, genetic information, diagnoses and treatments. It enables clinicians to describe the course of disease and to provide additional context about abnormal clinical findings (Figure 2). The Phenopacket Schema does not require the use of specific ontologies but it does strongly recommend the use of the HPO for representing phenotypic features of rare disease (47).

The Phenopacket schema is designed to support a wide range of use cases, including rare-disease diagnostics and integration within clinical laboratories, journals, data repositories, patient registries, EHRs and knowledge bases. (48). Software that supports diagnostic genome sequencing, including Exomiser, LIRICAL, Phen2Gene and SvAnna, can take phenopackets as input files (49–51), and we encourage all users of HPO to adopt phenopackets for data exchange.

### Electronic health record data interoperability

Common Data Models (CDMs) such as the Observational Medical Outcomes Partnership (OMOP) (52), are instrumental in making clinical data useful for research. CDMs have facilitated the flow of phenotypic data into and out of the Electronic Health Record (EHR), thereby enhancing genomic research and precision medicine. A previous effort systematically mapped terms in OMOP to OBO ontologies to facilitate deep phenotyping using EHR data (53). Manual and automated methods were used to map 92 367 conditions, 8611 drug ingredients and 10 673 measurement results from OMOP to OBO ontologies including Mondo, ChEBI and HPO, covering 68–99% of concepts used in clinical practice when examined across 24 hospitals. The clinical utility of the HPO mappings were assessed by comparing them to an existing set of manually validated mappings between the HPO and ICD (54) when used to identify undiagnosed rare disease patients from the All of Us Research project. Results of this analysis revealed that the OMOP2OBO HPO mappings performed as well as the manually validated mappings to identify relevant patients while using less query time and fewer clinical concepts. These mappings have also been employed for use cases such as applying ML to extract knowledge from EHR data. Data-driven feature engineering in ML models uses each distinct concept as a feature ignoring the fact that there are sometimes hundreds of concepts for the same or very similar features, and ignoring knowledge about relationships among concepts. HPO provides a pre-model data reduction of features that aids in the explainability and interpretability of ML features by consolidating related concepts or indicated known relationships among them. A recent effort extracted phenotypic features for long COVID patients, and applied ML to define subtypes of long COVID (34,55).

Health Level Seven (HL7) is the world's preeminent health data standards organization and its FHIR product family's rapid adoption worldwide has made it a universal transport platform for health data that can be constrained or extended to meet the business requirements of any health data exchange. HPO and Mondo are both registered within the HL7 Terminology Authority, whose role is to make terminologies created by external organizations available for use in any HL7 published product and to date they have been used in several HL7 FHIR Implementation Guides (IGs). Specifically relevant is the Vulcan Accelerator for Phenotypic Data, which is maturing an IG to extract content to populate Phenopackets for submission to clinical labs, de-identified content for research and other exchange purposes. This IG is complementary to and synergistic with the Clinical Genomics Working Groups’ IG focused on putting the results of variant interpretation back into the EHR. Together, the two enable genomic precision medicine, encoded using the HPO, within the EHR.

## Conclusion

The HPO has been under active development since its initial publication 15 years ago (3). The HPO has benefited enormously from contributions from members of the community to revise and extend specific clinical areas of the ontology. We welcome suggestions about additional areas of medicine whose representation in the HPO would benefit from targeted improvement. We have presented translations of the HPO and internationalization of the HPO website, workshops and additional terms in several areas of medicine, applications of HPO to the annotation and analysis of COVID-19, and integration of HPO with EHR resources and with the GA4GH Phenopacket Schema. The use of a standardized vocabulary for describing detailed phenotypes across different languages and in many different research and clinical settings supports interoperability, increases the value of clinical data by providing a clear and consistent semantic system to represent it, and facilitates the use of phenotypic information in informing clinical decision-making, bringing us closer to the goal of precision medicine.

## Data Availability

**Human Phenotype Ontology**: https://hpo.jax.org/: Files available for download include the main ontology file in OBO, OWL and JSON formats (see Download|Ontology); the main HPOA file, phenotype.hpoa (see Download|Annotation). **HPO translations and profiles:** https://obophenotype.github.io/hpo-translations/ **Chinese HPO**: https://www.chinahpo.net/ **IMPACT initiative**: https://genomica-impact.es **ENOD program**: https://www.ciberer.es/en/transversal-programmes/scientific-projects/undiagnosed-rare-diseases-programme-enod) **Spanish Network for Research on Rare Diseases (CIBERER)**: https://www.ciberer.es/en **International edition of HPO**: http://purl.obolibrary.org/obo/hp/hp-international.owl **Babelon - A simple standard for managing ontology translations and language profiles**: https://github.com/monarch-initiative/babelon **LinkML**: https://linkml.io/ **Language-specific versions of the HPO**: e.g. http://purl.obolibrary.org/obo/hp/translations/hp-fr.owl for French **Tabular files with translations of labels and definitions**: e.g. http://purl.obolibrary.org/obo/hp/translations/hp-fr.babelon.tsv for French **Language-specific synonyms**: e.g. http://purl.obolibrary.org/obo/hp/translations/hp-fr.synonyms.tsvfor French **GitHub**: https://github.com/obophenotype/human-phenotype-ontology **Change logs**: https://github.com/obophenotype/human-phenotype-ontology/tree/master/src/ontology/reports **Instructions for contributing to the HPO** are available at https://hpo.jax.org/app/help/collaboration **Monarch Initiative**: https://monarchinitiative.org/ **Ontology Access Kit (OAK)**: https://github.com/INCATools/ontology-access-kit **OMOP2OBO**: https://github.com/callahantiff/OMOP2OBO **Mondo Ontology:** https://mondo.monarchinitiative.org/ **Medical Action Ontology**: https://github.com/monarch-initiative/MAxO **Ontology of Biological Attributes (OBA):** https://github.com/obophenotype/bio-attribute-ontology **Gene Curation Coalition (GenCC)**: https://thegencc.org/ **fenominal**: https://github.com/monarch-initiative/fenominal. **SimulConsult**: https://simulconsult.com/ **PhenoTips**: https://phenotips.com/ **Face2Gene**: https://www.face2gene.com/
